# Deep Eutectic Systems: A Game Changer for Marine Bioactives Recovery

**DOI:** 10.3390/md23050211

**Published:** 2025-05-16

**Authors:** Sandro Amador, Alice Martins, Margarida Matias, Rui Pedrosa, Susete Pinteus

**Affiliations:** 1 Instituto de Tecnologia Química e Biológica António Xavier (ITQB), NOVA University of Lisbon, Av. da República, 2780-157 Oeiras, Portugal; 2 CENIMAT/I3N, Department of Materials Science, NOVA School of Science and Technology, NOVA University of Lisbon, 2829-516 Caparica, Portugal; 3 MARE-Marine and Environmental Sciences Centre/ARNET-Aquatic Research Network, ESTM, Polytechnic University of Leiria, 2520-630 Peniche, Portugal; margarida.matias@tus.ie (M.M.); rui.pedrosa@ipleiria.pt (R.P.); 4 LIFE-Health and Bioscience Research Institute, Technological University of Shannon, Moylish Park, V94 E8YF Limerick, Ireland

**Keywords:** blue bioeconomy, deep eutectic systems, marine-derived compounds, new-generation solvents, sustainable extraction technologies

## Abstract

The extraction of bioactive compounds from marine natural products has gained increasing attention due to their diverse applications, such as in pharmaceuticals, nutraceuticals, and cosmetics. Yet, low extraction yields and toxicity associated with common solvents are a major bottleneck. Deep eutectic solvents (DESs) and natural deep eutectic solvents (NADESs) have emerged as promising green alternatives to conventional organic solvents, offering advantages such as biodegradability, greater environmental and economic sustainability, low toxicity, and enhanced extraction selectivity. This review provides a comprehensive analysis of the principles, physicochemical properties, and applications of DESs/NADESs to obtain bioactive compounds from marine organisms. Among the most recent works, it is possible to verify the success of NADESs to extract carrageenan from the seaweed *Kappaphycus alvarezii*; pigments from *Palmaria palmata*; *and* polyphenols and proteins from different brown seaweeds. NADESs have also shown high potential to extract other valuable compounds from marine by-products, such as chitin from crabs and shrimp shells, and also lipids and proteins from different fish species and protein rich extracts from tilapia viscera. The challenges for DESs/NADESs use at industrial scale are also discussed, and success cases are revealed, highlighting their potential as game changers for extracting bioactive compounds from marine organisms and driving the development of innovative biotechnological products.

## 1. Introduction

The United Nations’ 2030 Sustainable Development Goals emphasize the importance of optimizing the use of existing resources while responsibly exploring untapped potential. The blue economy sector is fundamental to Europe’s economic growth, offering vast opportunities for innovation and prosperity [1,2,3]. Although the marine environment offers a wealth of high-value materials for developing innovative products and biotechnologies, significant progress is still needed to address the challenges of environmental sustainability, safety, and large-scale industrial production. To align with international sustainability goals, it is crucial to transition towards eco-friendly extraction technologies, such as green solvents, where deep eutectic systems offer a promising breakthrough for the industry.

### 1.1. Deep Eutectic Systems

The use of deep eutectic systems for extracting bioactive compounds from marine sources is an emerging and promising area of research. Previously, several studies showed the possibility of using ionic liquids (ILs) as alternative solvents for green technologies, but these present notable disadvantages. ILs are highly tunable solvents with an ever-growing presence and range of applications, but, in essence, they are organic salts with low melting points [4], characterized by poor biodegradability and sustainability while exhibiting toxicity at multiple levels, including in marine ecosystems. In 2003, Abbot et al. [5] tried to surpass these limitations and develop greener solvents, ultimately leading to a new category, deep eutectic solvents (DESs). Subsequently, a key property of deep eutectic systems was shown in the design of a new choline chloride/urea eutectic mixture with a melting point of 12 °C, much lower than both its constituents [5,6,7,8,9].

Deep eutectic solvents (DESs) are a class of eco-friendly, non-toxic, and biodegradable solvents formed by mixing two or more components, typically a hydrogen bond donor (HBD) and a hydrogen bond acceptor (HBA), which interact through hydrogen bonding to create a eutectic mixture with a melting point lower than that of its individual components [6]. Allying the multiple combination possibilities of HBA and HBD with their easy preparation, compliance with green synthesis, possible biocompatible applications and overall industry potential, the field of deep eutectic systems is expected to continue to grow throughout the years [7,10].

Usually, binary DESs are the most common, but they are not the only category of DES. In 2011, Gutiérrez et al. designed some of the first ternary DESs based on resorcinol, 3-hydroxypyridine, and choline chloride [11], while in 2014, Zhao et al. proved the possibility of producing ternary DESs based on mixtures of different fatty acids. This allowed for the assessment of the ternary eutectic point of ternary DESs [12].

Overall, DESs are highly versatile green solvents due to their inherent low volatility, sustainability, and tunability, since each DES property can be tuned by changing the compounds that act as HBAs and HBDs. For the past two decades, the number of compounds for DES design has been exponentially increasing, as there are several possible combinations and applications of these green solvents, to a point where a clear evolution can be traced [13].

### 1.2. Natural Deep Eutectic Systems

Natural deep eutectic systems—NADESs—have gained significant attention due to their biocompatibility, sustainability, and efficiency in various applications, including green chemistry, pharmaceuticals, food science, and biotechnology. One of the primary advantages of NADESs is their eco-friendliness and biodegradability. Being derived from natural resources, they are proven to be safe for both human health and ecosystems due to their safe environmental degradation, reducing chemical pollution [4]. Another major advantage of NADESs is their high solubility capacity. These solvents can dissolve a wide range of hydrophilic and hydrophobic compounds, including bioactive natural products, which are often poorly soluble in water or traditional solvents. This makes them highly valuable for pharmaceutical, nutraceutical, and cosmetic applications, such as the extraction of antioxidants, phenylpropanoids, polyphenols, and flavonoids from plants [7,14,15], or of other added-value hydrophilic or lipophilic compounds from seaweeds [16,17].

**Figure 1:**
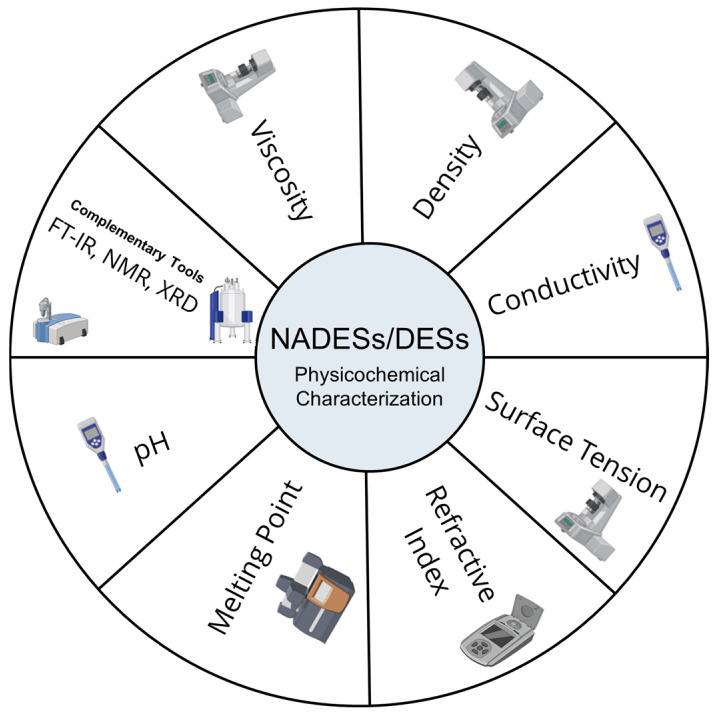
Main tools to achieve DES/NADES physicochemical characterization. (Icons provided by BioRender (www.biorender.com)).

The physicochemical properties of NADESs, such as their molar ratio, melting point, pH, density, viscosity, conductivity, refraction index, and polarity, were described, in 2022, by Freitas et al. [18]. The same authors performed a prediction of NADES chemical structure by using complementary tools such as NMR, FTIR, molecular modeling studies, molecular dynamics simulations, and quantum chemical calculations to characterize the NADESs used in a green extraction of cork bioactive compounds. These techniques (Figure 1) allow for the deep physicochemical characterization of DESs/NADESs, independently of their composition and final purpose.

The presence of water, even in small amounts, significantly influences the physicochemical and functional properties of NADESs. It has been shown that water can modulate parameters such as viscosity, density, surface tension, pH, and even antimicrobial activity. A recent study by Pozharitskaya et al. [19] systematically evaluated lactic-acid-based NADESs diluted with water, and reported that up to 40% water content led to a linear decrease in density and refractive index, a significant drop in viscosity, and a statistically significant increase in pH, without the complete disruption of the hydrogen bonding network. Importantly, they also observed that water content affected antimicrobial activity, which was decreased on average by 2–8 times after the addition of 40% water. It was found that pH has a significant impact on antimicrobial activity, with low pHs associated with higher antimicrobial activity. These findings underscore the importance of water content as a key factor in tailoring NADES properties for specific applications in extraction and bioactivity.

NADESs were first described by Choi et al. (2011) as a possible explanation for undergoing cellular metabolism and the possible existence of an alternative solvent to water and lipids, considering that compounds such as sugars, amino acids, and organic acids were present in several cells in high amounts with no clear metabolic explanation [20]. Despite their polarity, several DESs were developed based on these molecules and shown to have increased solubility compared to water [20]. However, there was a paradigm shift in 2015, with the advent of the first reported hydrophobic deep eutectic systems [21]. Hydrophobic deep eutectic systems (HDESs) were described by these authors as new extractants for the recovery of volatile fatty acids from aqueous solutions, based on decanoic acid and quaternary ammonium salts. Currently, HDESs have been extensively described in the literature, with several combinations of HBAs and HBDs, with the added benefit of using natural compounds for their design, broadening not only the library of HDESs but the concept of hydrophobic natural deep eutectic systems (HNADESs). Several bioactive and commercially available compounds are hydrophobic in nature, such as terpenes, essential oils, and fatty acids, hence, their use as compounds in HNADES design.

Some examples include menthol-based HNADESs (menthol + fatty acids (e.g., lauric acid, stearic acid)) for the extraction of non-polar compounds like carotenoids and essential oils [22]; thymol-based HNADESs (thymol + fatty acids (e.g., myristic acid, palmitic acid)), for pharmaceutical formulations and food preservatives [7,23]; camphor-based NADESs for the cosmetic industry [24], and fatty acid-based HNADESs (oleic acid + long-chain alcohols (e.g., dodecanol)) for drug delivery systems [25,26].

## 2. Materials and Methods

Literature Search Strategy All the information gathered in this scientific document was obtained through searches on the following databases: PubMed, Scopus, Web of Science, ScienceDirect, and Google Scholar.

Search Terms: Deep eutectic solvents; marine bioactive compounds; green extraction approaches; natural deep eutectic solvents; NADES; deep eutectic solvents to obtain seaweed compounds.

Timeframe: The search was focused on studies published from 2019 to 2025 to focus on recent developments.

Exclusion Criteria: Non-peer-reviewed sources, conference abstracts, and studies unrelated to the topic were excluded.

## 3. Current Applications of Deep Eutectic Systems

**Figure 2:**
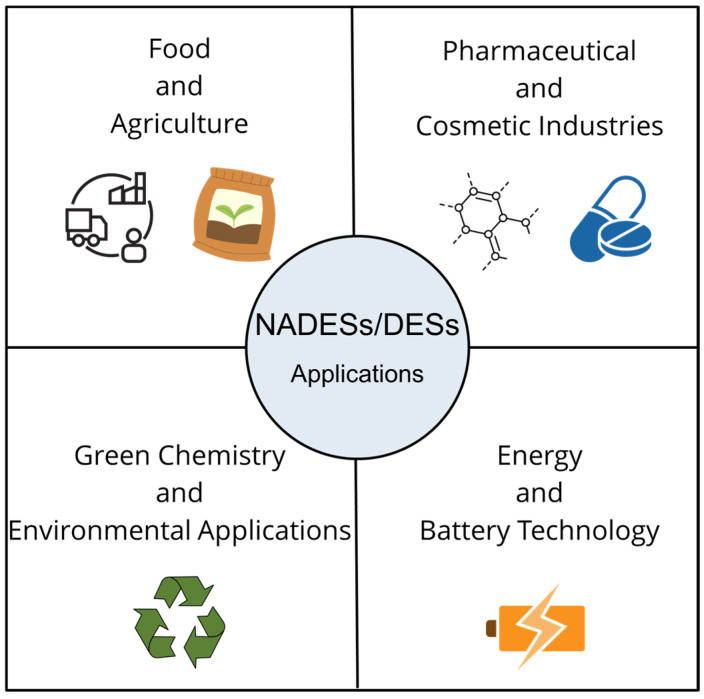
The industrial sectors that can benefit from the application of DESs/NADESs.

Comprehensive reviews on the application of deep eutectic systems (DESs/NADESs) to extract bioactive compounds from natural matrices, particularly from plants and agro-industrial by-products, have been recently published by several authors [27,28,29,30,31,32,33,34,35,36,37,38,39]. Overall, such reviews focus on aspects related to DES/NADES composition (HBA and HBD selection, molar ratio); modern extraction methodologies, in particular Microwave-Assisted Extraction (MAE) and Ultrasound-Assisted Extraction (UAE); and factors affecting extraction efficiency such as temperature, pH, extraction time, and solid-to-solvent ratio. The objective is to target bioactive compounds (bioorganic acids, fatty acids, carbohydrates, polyphenols, flavonoids, alkaloids, sterols, terpenes, saponins, pigments, polysaccharides, proteins, vitamins, amino acids, etc.) for different final applications, such as in the cosmetic, food, feed, biotechnology, agricultural, and pharmaceutical industries (Figure 2). Recently, the effects of selected extraction methods and NADESs on the recovery of active principles from *A. elata* were reported. The modern method of vibrocavitation-assisted extraction was considered as the most effective [40].

However, the use of DESs/NADESs as extraction media is not the only possibility for these systems, with further applications including their use in key industrial recycling processes. In 2021, a study by Pestana et al. [41] assessed the possibility of using NADESs in the recycling of polyethylene terephthalate (PET) industrial waste. Here, a NADES based on thymol and carvacrol was able to completely dissolve PET without degradation and with the retention of key properties, ensuring the total recovery of the NADES with later precipitation.

In recent years, there has been growing interest in the use of NADESs for various biomedical applications. One notable area is cryopreservation, where NADESs are being investigated as alternative cryoprotectants due to their low toxicity and compatibility with mammalian cell lines [42]. In addition, NADESs have also demonstrated potential in stabilizing proteins, such as trypsin [43], which is essential for maintaining protein activity and function during storage and handling. Despite these promising developments and the increasing attention on NADESs in the biomedical field, there remain many unanswered questions and opportunities for further research [44,45].

There are distinct variables when comparing the design phases of bioactive DESs compared with those of DESs for extraction purposes. While a DES for an extraction procedure will be tailored with compounds that can achieve a stable supramolecular structure and show a greater physicochemical affinity for the extracted molecules, for a bioactive DES there are two possible design scenarios. Either an active pharmaceutical ingredient (API) is incorporated into an existing stable DES, or the API can act as a component/counterpart to produce the bioactive DES, introducing relevant bioactivity into a stable supramolecular structure at the same time.

Menthol, thymol, and several organic fatty acids have been used repeatedly in several eutectic systems for diverse biomedical applications, from wound healing to anticancerogenic activity [23,46,47].

**Figure 3:**
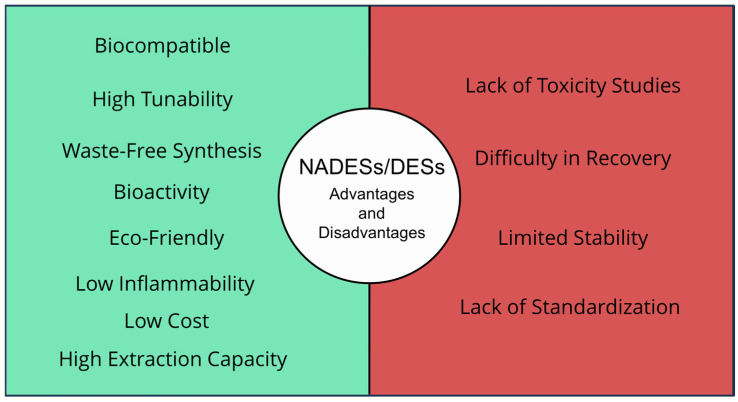
The main advantages and disadvantages of DESs/NADESs.

While DESs/NADESs have various applications, it is crucial to evaluate their advantages and disadvantages (Figure 3).

Particular attention should be paid to their limitations, especially concerning potential toxicity and long-term stability [48].

### Toxicity of DESs/NADESs

The issue of DESs toxicity remains a topic of active discussion and research. Although the number of comprehensive studies is still limited, several key factors have been identified that influence toxicity. These include the specific starting compounds used to formulate the DESs or NADESs, the molar ratios in which these compounds are combined, and the interactions that occur between them. Importantly, when it comes to NADESs, the natural origin of a compound does not necessarily guarantee a low toxicity. In fact, organisms can be more sensitive to mixtures of natural components than to the individual substances themselves, due to possible synergistic effects that enhance toxicity [49,50].

Early studies have already demonstrated that such synergistic toxicity can occur [50], sometimes resulting in harmful consequences for living organisms. This underscores the urgent need for further research that systematically evaluates the toxicity of DESs and NADESs mixtures. Additionally, for applications where these solvents may come into direct or indirect contact with the environment, it is essential to incorporate ecotoxicological assessments into future studies.

While this area has garnered increasing attention in recent years, significant room for improvement remains. A comprehensive evaluation encompassing atmospheric, aquatic, and terrestrial ecosystems is necessary, moving beyond the current predominant focus on aquatic environments alone. By integrating the determination of key toxicological parameters, future studies can offer a more robust and holistic understanding of DESs toxicity [48,49,51].

## 4. Extraction of Bioactive Ingredients from Marine Biomass

As referred above, while the application of DESs in extracting bioactive compounds from terrestrial biomass is fully described, the use of such systems to obtain added-value compounds from marine sources is an emerging area of research. Effectively, specific reviews focusing solely on the use of DESs/NADESs to extract marine biomass [52,53,54,55,56,57] are scarce, although some reviews reporting the use of eutectic systems in natural product extraction also include marine-derived compounds [58,59,60]. Overall, these reviews underscore the emerging interest in utilizing DESs/NADESs for the extraction of valuable compounds from marine biomass.

Studies on different DES/NADES-based extraction methods for high-value compounds (e.g., phycobiliproteins, PUFAs) from the microalgae *Porphyridium* sp., combining UAE (Ultrasound-Assisted Extraction) and COSMO-RS (Conductor-Like Screening Model for Real Solvents) modeling to predict cell wall solubility in various DESs, have been recently reported [52,53,54,55,56,57]. According to these authors, in some cases, although the extraction yield was lower than with aqueous extractions, the NADES extracts exhibited enhanced stability and preserved antioxidant activity over time.

In 2024, Roy et al. [53] focused on the extraction and stabilization of marine carotenoids from several species of cyanobacteria, micro- and macroalgae, and marine animals for potential applications in the food, pharmaceutical, and cosmetic industries. Green extraction techniques that used supercritical carbon dioxide and NADESs were highlighted and compared to conventional extraction solvents. The use of DESs to extract marine compounds with pharmaceutical interest was also reported in 2022 [56], providing an in-depth analysis of DES properties, versatility, production methods, and their applications in extracting bioactive compounds from various natural sources, including marine environments. The authors reported the progress made on DESs for processing crustacean and fish by-products and seaweeds for the extraction of polysaccharides, proteins, and pigments, as well as their use to create functional materials. The main issues concerning DESs properties, extract purification, toxicity, and biodegradation were also stated. The authors concluded that, despite some challenges, DESs are promising systems for the processing of marine biomass. The undeniable advantages of NADESs make them promising, economically viable, and environmentally friendly alternatives for different extraction applications to supply safe extracts for the food, pharmaceutical, and cosmetics industries.

A short review on the use of NADESs to extract other added-value compounds from microalgae such as *Dunaliella salina* (β-carotene), *Nannochloropsis gaditiana* (docosahexaenoic acid (DHA), eicosapentaenoic acid (EPA), α-linolenic acid (ALA), arachidonic acid (ARA), γ-linoleic acid (GLA), *Lyngbya majuscule* (microcolin-A), and *Haslea ostrearia* (tocopherols) was also presented by Mehariya et al. [57]. More recently, Vo et al. [58] reviewed novel extraction techniques (UAE; MAE—Microwave-Assisted Extraction; EAE—Enzyme-Assisted Extraction; SFE—Supercritical Fluid Extraction) and the use of green solvents, including DESs/NADESs, to recover bioactive compounds from the macroalgae *Arctic Fucus vesiculosus* (phlorotannins), *Sargassum muticum* (phenolic compounds), and *Hypnea flagelliformis* (phenolics), and from the microalgae *Chromochloris zofingiensis* (canthaxanthin) and *Spirulina platensis* (pigments). For such marine biomass, the extraction conditions were also summarized regarding solid/liquid ratio, time, temperature, DESs/NADESs molar ratios, and extraction yields. According to Quitério et al. [55], eutectic solvents are suitable for extracting bioactive compounds from seaweeds by using several advanced techniques such as PLE (Pressurized Liquid Extraction), MAE, and UAE, being compatible with food, cosmetic, and pharmaceutical uses due to their high biodegradability and lower toxicity.

The use of DESs to extract carbohydrates from algal biomass (*Fucus vesiculosus*, *Saccharina japonica*, *Sargassum horneri*, *Kappaphycus alvarezii*) was reviewed by Liu et al. [59], exploring factors such as DES molar ratio and conditions that influence the behavior of DESs in carbohydrate (fucoidan, alginate, and carrageenan) extraction. In addition, the extraction of *k*-carrageenan from *Kappaphycus alvarezii* was carried out by using either DESs prepared by the complexation of choline chloride with urea, ethylene glycol, and glycerol or by water as a standalone extraction media [54]. Interestingly, for the best DES formulation, the *k*-carrageenan yield achieved the highest value when combined with water (60%) due to the higher affinity of the DES charges towards *k*-carrageenan, which are absent in pure water, thus showing lower extraction efficiency in that scenario. Another work [60] provided an overview of DESs with an emphasis on their unique physicochemical properties that make them superior green solvents due to their low or non-toxicity, biodegradability, easy preparation, renewability, and tailorable properties. The importance of DESs properties, particularly viscosity, polarity, molar ratio, and water addition on the DESs extraction performance was discussed. In addition, advances in the DESs extraction of marine-derived biomolecules were presented, namely, the extraction of polysaccharides from seaweeds (*Saccharina japonica*, *Sargassum horneri*; *Fucus vesiculosus*), astaxanthin from the microalgae *Haematococcus pluvialis*, and free fatty acids from *Spirulina* sp., as well as chitin from lobster shells and proteins from sardine processing residues. Also, a study performed in 2022 provided insights into using other hydrophobic NADESs based on oleic acid for the exclusive extraction of astaxanthin from *H. pluvialis*, with a 60% recovery rate [61]. Another study investigated the extraction of fucoxanthin from the algae *Tisochrysis lutea* using HNADESs, showing an increase in yield and also in keeping the added-value compound stable within the HNADESs for 11 days [62].

An update (2019–2024) of works involving the extraction of marine-derived compounds by using DESs/NADESs is summarized in Table 1.

A brief analysis of Table 1 reveals that micro- and macroalgae are the predominant biomass extracted with eutectic systems, followed by residues resulting from marine animals’ food industry processing. Lipophilic molecules such as saturated and polyunsaturated fatty acids, carotenoids—particularly astaxanthin—as well as hydrophilic ingredients—e.g., proteins, phenolics, and carbohydrates, including complex polysaccharides such as chitin, fucoidan, and carrageenan—are the main target molecules. Regarding the hydrogen bond acceptors (HBAs), choline chloride arises as the most used HBA, while alcohols (glycerol, propanediol, ethylene glycol), organic acids (malic, levulinic, oxalic, citric, acetic, lactic acids), sugars (glucose, fructose), and urea appear as the most used hydrogen bond donors (HBDs).

From a sustainability point of view, the work performed with DESs to obtain bioactive compounds from marine by-products is essential. These by-products, such as fish scales, viscera, shells, and bones, are often discarded or underutilized, despite being rich in valuable bioactive compounds. The use of DESs in extraction not only minimizes waste but also promotes a circular economy by valuing waste. As consumer awareness increases, industries are increasingly turning to untapped resources like marine by-products to develop innovative products while addressing their impact on the environment. These materials seem to be rich in bioactive molecules such as omega-3 fatty acids, peptides, and chitin, which can be used in nutraceuticals, pharmaceuticals, and cosmetics.

## 5. Commercial Products Utilizing DESs

DESs have been integrated into various commercial products across multiple industries, demonstrating their biotechnological potential and versatility. The following are some example products from the pharmaceutical industry:

- EMLA^®^ Cream: This topical anesthetic combines lidocaine and prilocaine in a eutectic mixture, enhancing skin penetration and providing effective local anesthesia. Approved by the FDA in 1992, EMLA^®^ Cream is widely used for minor surgical procedures and needle insertions [88].
- SYNERA^®^ Patch: Utilizing a eutectic mixture of lidocaine and tetracaine, this topical patch offers localized pain relief. Its self-heating technology facilitates drug delivery, making it suitable for various dermatological procedures [89].

These examples illustrate the practical applications of DESs in commercial products, highlighting their relevance to delivering highly valued products, with an impact on society. While their use to obtain bioactive ingredients from marine organisms has not been documented at an industrial scale, this new technology will certainly be an innovative solution for the development of new marine-derived products. Marine-derived bioactive compounds often suffer from issues such as low solubility and bioavailability. DESs present a unique solution for this purpose, as these solvents can be utilized in encapsulation technologies to protect and deliver these compounds more efficiently [90]. The ability of DESs/NADESs to form stable complexes with bioactive molecules is, therefore, advantageous for creating novel delivery systems not only for pharmaceuticals, but also for producing functional foods and functional greener cosmetics derived from marine resources. The future lies in improving the interaction between DESs/NADESs and the encapsulated materials to ensure controlled release and stability.

## 6. Conclusions and Future Directions

The use of DESs to obtain valuable ingredients from marine resources aligns with the growing trend toward sustainable and environmentally friendly practices in the industry. While DESs are promising, their commercial-scale use for marine products still faces challenges:

- The lack of standardized protocols for the use of DESs in different industries:

Different formulations of DESs can yield different results, and there is still much to be explored in terms of the best DES combinations for extracting or processing specific marine compounds. There is a need for further research to understand the interaction of various DESs with marine matrices, as well as to standardize their use in industrial applications.

- The long-term stability and reusability of DESs:

DESs can be reutilized after one extraction, but this depends on their stability under different processing conditions [91]. Research into the reusability of DESs and their resistance to degradation is essential for enhancing their economic viability in industrial applications.

- The commercialization of marine products obtained with DESs requires regulatory approval:

The toxicity of DESs and NADESs remains an active area of research, with key factors such as component selection, molar ratios, and synergistic interactions significantly influencing their effects. The current evidence highlights that a natural origin does not guarantee a low toxicity, and mixtures may pose greater risks than individual components. Therefore, comprehensive and systematic ecotoxicological assessments across diverse ecosystems are urgently needed to ensure the safe application of these solvents.

The development of clear safety guidelines and certification processes for DES-based products will be essential to ensure their acceptance in global markets. Marine-based applications, such as food or pharmaceuticals, are highly regulated, and it is important to investigate how DESs can meet these stringent regulatory requirements.

In conclusion, deep eutectic systems are a breakthrough in the extraction of bioactive compounds from nature and marine environments alike. Their customizable nature and ability to enhance extraction efficiency make them valuable tools for applications. The studies reviewed in this manuscript demonstrate that DESs/NADESs can be tailored to extract specific marine biomolecules, ranging from polysaccharides and polyphenols to carotenoids and proteins. However, further research is needed to address challenges related to their limitations, such as possible toxicity, biodegradability, and industrial scalability. Future work should focus on optimizing DESs/NADESs formulations, developing purification strategies, exploring their recyclability and reutilization, and assessing the long-term environmental impact of these solvents on marine and terrestrial environments. With its unique advantages, DESs/NADESs application could revolutionize green extraction technologies and contribute to a more sustainable blue bioeconomy if its limitations are assessed and overcome.

## Figures and Tables

**Table 1:** Marine-derived compounds extracted with DESs/NADESs.

Marine Organisms	DES/NADES	Biomolecules	References
Macroalgae			
*Ascophyllum nodosum*	Betaine:lactic acid:H_2_O (1:1:2) Choline chloride:malic acid:H_2_O (2:1:1)	Phlorotannins	[16]
*Codium tomentosum*	Menthol:octanoic acid	Phytosterols	[63]
*Fucus vesiculosus*	Choline chloride:1,4-butanediol (1:5)	Sugars	[59]
*Fucus vesiculosus*	Lactic acid:glucose:H_2_O (5:3:1)	Trace Metals	[64]
*Fucus vesiculosus*	Betaine:lactic acid:H_2_O (1:1:2) Choline chloride:malic acid:H_2_O (2:1:1)	Phlorotannins	[16]
*Fucus vesiculosus*	Lactic acid:choline chloride (3:1) Lactic acid:glucose:H_2_O (5:1:3)	Ascorbic Acid Fucoxanthin	[17]
*Gelidium corneum*	L-lactic acid:fructose (7:1)	Phenolics	[65]
*Kappaphycus alvarezii*	Choline chloride:urea (1:2) Choline chloride:ethylene glycol (1:2) Choline chloride:glycerol (1:2) 10% hydrated choline chloride:urea (1:2) 10% hydrated choline chloride: ethylene glycol (1:2) 10% hydrated choline chloride: glycerol (1:2)	Carrageenans	[59]
*Palmaria palmata*	Glycerol:glucose (2:1/50%) Choline chloride:glycerol (1:2/60%)	R-Phycocyanin Allophycocyanin B-Phycoerythrin Proteins Sulfated Polysaccharides Polyphenols	[66]
*Saccharina japonica*	Choline chloride:glycerol (1:2)	Fucoidan Alginate	[59]
*Saccharina latissima*	Betaine:1,3-butanediol (1:1)	Polyphenols	[67]
*Saccharina latissima*	Choline chloride:oxalic acid; choline chloride:urea; choline chloride:levulinic acid; betaine:urea:water	Proteins	[68]
*Saccharina latissima*	Menthol:levulinic acid	Chlorophyll Fucoxanthin	[69]
*Sargassum* *muticum*	L-lactic acid:fructose (5:1) L-lactic acid:glucose (5:1) L-lactic acid:sodium acetate (7:1)	Phenolics	[65]
*Sargassum* *muticum*	Proline:propylene glycol Proline:1,2-butanediol Choline:citric acid	Phenolics	[70]
Microalgae			
*Arthrospira platensis*	Octanoic acid:1,3-propanediol (5:1) Octanoic acid:decanoic acid:1,3-propanediol (3:1:1)	Free Fatty Acids	[71]
*Chlorella vulgaris*	Choline chloride:acetic acid (1:2) Choline chloride:urea (1:2)	Lipids Carotenoids Proteins Carbohydrates Phenolics	[72]
*Chlorella vulgaris*	Choline chloride:1,2-butanediol (1:4) Choline chloride:ethylene glycol (1:2) Choline chloride:glycerol (1:2)	Carotenoids Phenolics	[73]
*Engineered* *Chlamydomonas reinhardtii*	Menthol:acetic acid (1:1) Menthol:lactic acid:H_2_O (3:3:1) Menthol:caprylic acid (1:1)	Astaxanthin	[74]
*Haematococcus lacustris*	Menthol:acetic acid (1:1) Menthol:lactic acid:H_2_O (3:3:1) Menthol:caprylic acid (1:1)	Astaxanthin	[74]
*Haematococcus* *pluvialis*	Oleic acid:thymol (1:1)	Astaxanthin	[60]
*Nannochloropsis gaditana*	Choline chloride:ethylene glycol	Eicosapentaenoic Acid (EPA)	[75]
*Neochloris texensis*	Choline chloride:acetic acid (1:2) Choline chloride:urea (1:2)	Lipids Carotenoids Proteins Carbohydrates Phenolics	[72]
*Porphyridium cruentum*	Octanoic acid:1,3-propanediol (5:1) Octanoic acid:decanoic acid:1,3-propanediol (3:1:1)	Free Fatty Acids	[71]
*Scenedesmus protuberans*	Choline chloride:acetic acid (1:2) Choline chloride:urea (1:2)	Lipids Carotenoids Proteins Carbohydrates Phenolics	[72]
*Schizochytrium* sp.	Choline chloride:acetic acid (1:2) Choline chloride:urea (1:2)	Lipids Carotenoids Proteins Carbohydrates Phenolics	[72]
*Spirulina* sp.	Nonanoic acid:decanoic acid:lauric acid (3:2:1)	Fatty Acids	[60]
*Thalassiosira andamanica*	HBA:quaternary ammonium salt HBD:organic acids and alcohols	Fucoxanthin Chlorophyll Biosilica	[76]
*Tisochrysis lutea*	Thymol:dodecanoic acid (1.25:1)	Fucoxanthin	[62]
Mollusk			
*Haliotis discus Hannai Ino*	Choline chloride:ethylene glycol (1:3/25%)	Polysaccharides	[77]
Marine By-Products			
*Chionoecetes opilio* (crab shells)	Triethylbenzylammonium chloride:lactic acid (1:27)	Chitin	[78]
Codfish bones	Fructose:lactic acid (1:5) Urea:lactic acid (1:4)	Lipids Proteins	[79]
*Gadus morhua* (fish skin, Atlantic codfish)	Urea:propionic acid (1:2)	Collagen Hydrolisates	[80]
*Loligo vulgaris* (squid pens)	Potassium carbonate:glycerol	beta-Chitin	[81]
*Macruronus novaezelandaiae* (tissues of fish species)	Menthol:carvacrol Menthol:thymol	Lipids	[82]
Mussel meat	Fructose:lactic acid (1:5) Urea:lactic acid (1:4)	Lipids Proteins	[79]
*Oreochromis niloticus* (tilapia viscera hydrolysate)	Choline chloride:1,4-butanediol Choline chloride:glycerol Choline chloride:lactic acid Choline chloride:urea Betaine:propylene glycol	Protein-Rich Extracts	[83]
*Penaeus monodon* (Shrimp shells)	Choline chloride:glycerol (1:2)	Chitin	[84]
*Perna canaliculus*	Menthol:carvacrol Menthol:thymol	Lipids	[82]
*Perna canaliculus*	Menthol:lidocaine (1:1) Menthol:lidocaine (1:2)	Eicosapentaenoic Acid (EPA) Docosahexaenoic Acid (DHA)	[85]
*Polybius henslowii*	Choline chloride:malonic acid Choline chloride:lactic acid	alpha-Chitin	[86]
*Prionace glauca* (fish skins)	Citric acid:xylitol:H_2_O (1:1:10)	Collagen	[87]
*Trachurus declivis* (tissues of fish species)	Menthol:carvacrol Menthol:thymol	Lipids	[82]
Tuna vitreous humor	Fructose:lactic acid (1:5) Urea:lactic acid (1:4)	Lipids Proteins	[79]

## Abbreviations

The following abbreviations are used in this manuscript:

DES	Deep eutectic system/solvent
ILs	Ionic liquids
HBA	Hydrogen bond acceptor
HBD	Hydrogen bond donor
NADES	Natural deep eutectic system/solvent
HDES	Hydrophobic deep eutectic system/solvent
HNADES	Hydrophobic natural deep eutectic system/solvent
API	Active pharmaceutical ingredient
